# Serum CC-Chemokine Profiling in Alcohol-Associated Liver Disease: Focus on MIP-3α/CCL20 as a Biomarker of Disease Severity

**DOI:** 10.3390/diagnostics16162520

**Published:** 2026-08-10

**Authors:** Agnieszka Szczerbinska, Jacek Rolinski, Agata Surdacka, Halina Cichoz-Lach

**Affiliations:** 1 Doctoral School, Medical University of Lublin, 20-093 Lublin, Poland; 2 Department of Clinical Immunology, Medical University of Lublin, 20-093 Lublin, Poland; 3 Department of Gastroenterology and Hepatology with Endoscopy Unit, Medical University of Lublin, 20-090 Lublin, Poland

**Keywords:** alcohol-associated liver disease, CC-motif chemokines, MIP-3α/CCL20, MCP-1/CCL2, TARC/CCL17, biomarkers

## Abstract

**Background/Objectives:** The inflammatory response is central to the pathogenesis of alcohol-associated liver disease (ALD), yet currently used prognostic scores, including Maddrey’s modified discriminant function (mDF), Child–Turcotte–Pugh (CTP), MELD-Na and MELD 3.0, reflect hepatic synthetic and excretory function rather than the underlying immune alterations. Since CC-motif chemokines (CCLs) regulate immune cell trafficking and hepatic stellate cell activation, they warrant deeper investigation, as reports on their role in ALD remain limited. Therefore, the primary objective of this study was to evaluate the diagnostic and prognostic performance of selected serum CCLs, and to correlate their levels with disease severity and short-term survival in patients with ALD. **Methods:** Serum MIP-3α/CCL20, MCP-1/CCL2 and TARC/CCL17 were measured in 63 patients with ALD and 25 healthy controls using ELISAs. Chemokine concentrations were correlated with liver function tests, inflammatory indices and short-term (30-day) survival. The diagnostic and prognostic performances of individual CC chemokines were assessed by ROC analysis and by univariate and multivariable logistic regression. **Results:** MIP-3α/CCL20 was increased more than 11-fold in ALD versus healthy controls and reached an AUC of 0.978 for distinguishing the two groups in a case-control comparison (a proof-of-principle, case-control result which requires validation against other chronic liver diseases). It increased across Child–Turcotte–Pugh classes and correlated with all other prognostic scales. MCP-1/CCL2 was significantly elevated in the ALD group but did not discriminate ALD severity. TARC/CCL17 was significantly lower in ALD than in controls. On multivariable regression, MIP-3α/CCL20 was the dominant predictor of both ALD status and severe liver dysfunction. **Conclusions:** In patients with ALD, MIP-3α/CCL20 appears to bridge hepatic inflammation and hepatocellular failure and is a candidate exploratory biomarker of disease severity that requires further confirmation against other chronic liver diseases. The short-term mortality analysis was based on only seven events. This evaluation is exploratory and underpowered, and its findings should not be interpreted as definitive prognostic evidence. MCP-1/CCL2 does not stratify ALD severity. Decreased TARC/CCL17 concentrations point to a separate immunoregulatory failure and are presented as a hypothesis requiring mechanistic testing in pre-clinical models.

## 1. Introduction

Alcohol-associated liver disease (ALD) is a leading cause of liver-related death worldwide. About half of all liver-cirrhosis deaths and nearly one-fifth of deaths from hepatocellular carcinoma (HCC) are attributable to ALD [1,2]. The burden continues to rise alongside per-capita alcohol consumption and reflects post-COVID-19 pandemic shifts in drinking patterns [3,4]. The disease spans a continuum from simple steatosis through alcohol-associated hepatitis (AH) to progressive fibrosis and cirrhosis. Acute-on-chronic liver failure (ACLF) may complicate the course, with short-term mortality of 30–50% in the most severe cases [5,6]. Treatment options remain limited: corticosteroids for severe AH, and abstinence and liver transplantation for end-stage disease [7]. Although hepatocyte injury caused by ethanol and its oxidative byproducts is the initial event, dysregulated innate and adaptive immunity is now recognised as the principal driver of disease progression [8,9,10,11].

Chronic ethanol intake increases intestinal permeability and induces microbial dysbiosis, activating the gut–liver axis. Kupffer cells take up pathogen-associated molecular patterns (PAMPs), mainly lipopolysaccharide (LPS), via TLR4/CD14 and release inflammatory mediators through NF-κB and NLRP3 inflammasome signalling [10,12,13]. A parallel sterile inflammation is driven by damage-associated molecular patterns (DAMPs), including high-mobility group box 1 (HMGB1) released from dying hepatocytes, mitochondrial DNA and extracellular ATP [7,14,15]. The resulting cytokine and chemokine synthesis recruits monocytes, neutrophils and polarised T-cell subsets to the liver and activates resident hepatic stellate cells (HSCs) and liver sinusoidal endothelial cells, which trans-differentiate into myofibroblasts and amplify fibrogenesis [14,16,17]. Chemokine gradients link inflammation to fibrosis [18,19]. Since chemokine ligands are readily measured in peripheral blood, they have attracted interest as candidate biomarkers and therapeutic targets in chronic liver injury, particularly in ALD where liver biopsy is often contraindicated due to coagulopathy and thrombocytopenia [20].

Three CC-chemokines were chosen for the present study because each engages a distinct receptor and immune-cell compartment relevant to ALD. CCL2 (MCP-1) signals through CCR2 and recruits Ly6C^high^ monocytes that differentiate into pro-inflammatory and scar-associated hepatic macrophages (SAMs) [21,22,23]. Murine studies have shown that CCL2 contributes to alcohol-induced steatosis, increased cytokine synthesis and fibrogenesis, and that pharmacological CCL2 blockade ameliorates alcohol-related liver injury [24]. CCL20 (MIP-3α) is the only known high-affinity ligand for CCR6, a receptor expressed on Th17 lymphocytes, group-3 innate lymphoid cells, IL-17-producing γδ T cells and immature dendritic cells [25]. Kupffer cells and HSCs produce CCL20 in response to LPS, and serum CCL20 has been linked to fibrosis stage, portal hypertension and endotoxaemia in AH. In vitro, CCL20 induces a pro-inflammatory and pro-fibrogenic phenotype in HSCs [26,27]. The CCL20/CCR6 axis has also been implicated in hepatocarcinogenesis through Treg recruitment into the tumour microenvironment [28]. CCL17 (TARC) signals through CCR4 and is produced predominantly by mature myeloid dendritic cells. It recruits Th2 effector cells and Foxp3+ Tregs and therefore plays a dual role in immunoregulation and inflammation [29,30]. In chronic viral hepatitis and HCC, intrahepatic CCL17 contributes to Treg-dependent immune tolerance and impairs antiviral immunity [31,32], and it has been proposed as a marker of the transition from inflammatory to tolerogenic phases of liver disease. Its behaviour in ALD has not been characterised so far.

Despite this mechanistic rationale, clinical reports on CC-family chemokines in ALD are sparse, and current prognostic models—Child–Turcotte–Pugh (CTP), Model for End-Stage Liver Disease (MELD), MELD-Na and the modified Maddrey discriminant function (mDF)—do not incorporate inflammatory mediators that may influence ALD outcome [33,34]. We hypothesised that selected CC-chemokines (CCL20, CCL2 and CCL17) might be involved in the pathogenesis of ALD by mediating immune and inflammatory activation. The primary endpoint of our study was the comparison of circulating CC-chemokine concentrations in patients with ALD and healthy controls. Secondary endpoints included the following: (i) correlations of CC-chemokine blood levels with biochemical parameters of liver function and conventional inflammatory markers; (ii) evaluation of the diagnostic performance of individual CC-chemokines; and (iii) assessment of the prognostic accuracy of the selected CC-chemokines for severe liver dysfunction and short-term (30-day) survival. Our single-centre prospective observational study was conducted in the Department of Gastroenterology and Hepatology with the Endoscopy Unit of the Medical University of Lublin (Poland), with chemokine assays performed in cooperation with the Department of Clinical Immunology of the same institution. All procedures adhered to the principles of the Declaration of Helsinki, and ethical approval for the protocol was granted by the Bioethics Committee of the Medical University of Lublin on 28 April 2022 (reference number: KE-0254/96/04/2022).

## 2. Materials and Methods

The present analysis of CC-motif chemokines (CCL20, CCL2 and CCL17) was performed in the same prospective single-centre cohort and under the same study protocol described in detail in our companion paper on CXC-motif chemokines in ALD [35]. Readers are referred to that publication for full details. A short overview of the study protocol and the modifications specific to CCL measurement are reported below.

### 2.1. Study Population

The study enrolled 63 adult patients with ALD admitted to the Department of Gastroenterology of the Medical University of Lublin, Poland, and 25 healthy volunteers (88 participants in total). Patients were enrolled consecutively over a 14-month period. Inclusion required a clinical, biochemical and ultrasonographic diagnosis of ALD and self-reported active alcohol consumption confirmed by the AUDIT-C questionnaire (a score of ≥4 for men and ≥3 for women) [36]. Exclusion criteria were viral hepatitis B or C, autoimmune liver disease, metabolic dysfunction-associated steatotic liver disease (MASLD), hereditary liver disease, biliary-tract disease, active malignancy, HIV infection, ongoing corticosteroids, antibiotics, immunomodulators or blood transfusions within the 6 months before blood sampling, and clinically apparent infection at admission. All patients were screened for occult infection (full blood count with C-reactive protein, chest radiograph, urinalysis, procalcitonin and blood cultures where clinically indicated). Patients with ascites underwent diagnostic paracentesis, and spontaneous bacterial peritonitis was excluded. Patients with confirmed infection at admission were not enrolled. Healthy controls had no history of liver disease, alcohol misuse, chronic infection, malignancy or immune-mediated disorders, and had normal liver enzymes on screening. All participants provided written informed consent.

### 2.2. Clinical and Laboratory Assessment

Patients’ clinical history and physical examination were recorded at hospital admission. Severity of liver dysfunction/alcohol-associated hepatitis (AH) was graded using the CTP with class assignment A, B or C, MELD-Na, MELD 3.0 and mDF.

We applied the following definitions of liver disease severity:

(1) Severe liver dysfunction: (a) CTP class C; (b) MELD-Na ≥ 20; or (c) MELD 3.0 > 19.
(2) Severe alcohol-associated hepatitis (AH): mDF ≥ 32.

Their prevalence within the ALD cohort is presented in Table 1.

Routine biochemistry (alanine aminotransferase [ALT], aspartate aminotransferase [AST], gamma-glutamyl transferase [GGT], alkaline phosphatase [ALP], total bilirubin, albumin, international normalised ratio [INR], urea and creatinine), complete blood count (CBC) and C-reactive protein (CRP) were performed by the staff in the Central Laboratory at the University Hospital No. 4, Lublin, Poland. Composite inflammatory indices were calculated based on CBC results as follows: neutrophil-to-lymphocyte ratio (NLR = neutrophils/lymphocytes) and systemic immune-inflammation index (SII = platelets × neutrophils/lymphocytes). The Fibrosis-4 index (FIB-4) was calculated from admission values as (age [years] × AST [U/L])/(platelet count [10^9^/L] × √ALT [U/L]).

Patient short-term survival within 30 days was recorded as the study endpoint and ascertained through hospital, outpatient clinic or phone follow-up records.

### 2.3. Chemokine Measurements

Peripheral venous blood was collected into plain serum tubes within 48 h of admission, allowed to clot, centrifuged at 3000× *g* for 10 min and stored immediately in aliquots at −80 °C until analysis. Serum concentrations of MIP-3α/CCL20, MCP-1/CCL2 and TARC/CCL17 were measured using commercial sandwich enzyme-linked immunosorbent assay (ELISA) kits (R&D Systems™, Bio-Techne, Minneapolis, MN, USA), following manufacturers’ protocols. Specifically, the following R&D Systems Quantikine kits were used: Human CCL2/MCP-1 (Catalog # DCP00; calibrated range 31.2–2000 pg/mL; mean minimum detectable dose [MDD]: 1.7 pg/mL), Human CCL17/TARC (Catalog # DDN00; range: 31.2–2000 pg/mL; MDD: 7 pg/mL) and Human CCL20/MIP-3α (Catalog # DM3A00; range: 7.8–500 pg/mL; mean MDD: 0.47 pg/mL). Samples underwent a single freeze–thaw cycle to minimise analyte degradation. All standards and samples were assayed in duplicate. Serum samples were processed according to each kit insert, including the manufacturer-specified 2-fold pre-dilution in Calibrator Diluent RD6Q for the CCL2/MCP-1 assay. Optical density was recorded at 450 nm with wavelength correction at 540 nm using a VICTOR™3 microplate reader (PerkinElmer, Waltham, MA, USA). Concentrations were calculated using standard curves generated from recombinant chemokine standards supplied in each kit, based on a four-parameter logistic (4-PL) regression model. The analytical sensitivity, measurement range, and intra- and inter-assay coefficients of variation (<10%) were consistent with the manufacturer’s specifications. Samples with concentrations exceeding the upper limit of quantification were appropriately diluted and reanalysed. Assay linearity and matrix compatibility were confirmed by serial dilution of representative serum samples, yielding recoveries within 80–120% of expected values. All kits used in this study have been independently validated by the manufacturer for dilutional linearity and matrix effects in human serum indicating negligible matrix interference and acceptable parallelism with the standard curve. The laboratory staff were blinded to case/control status and severity of liver dysfunction. Because each participant was sampled once, within 48 h of admission, the design is cross-sectional with respect to biomarker measurement and cannot establish whether the chemokine changes precede or follow clinical decompensation.

### 2.4. Statistical Analysis

Statistical analyses were performed using MedCalc® Statistical Software version 23.5.2 (MedCalc Software Ltd., Ostend, Belgium; https://www.medcalc.org; 2026) [37]. The Shapiro–Wilk test revealed a non-normal distribution of all three chemokines, so non-parametric methods were used throughout. Continuous variables are reported as medians with interquartile ranges (IQRs). Two-group comparisons used the Mann–Whitney U test, with effect size reported as the rank-biserial correlation (r). Multi-group comparisons used the Kruskal–Wallis test with Bonferroni-corrected post-hoc pairwise testing, with effect size reported as epsilon-squared (ε^2^). Associations between continuous variables were examined by Spearman’s rank correlation, with partial correlations adjusted for age and sex. To control the false-discovery rate across the chemokine–marker correlation matrix, the Benjamini–Hochberg procedure was applied, and both raw *p*-values and adjusted q-values are reported. Diagnostic accuracy was assessed by ROC-curve analysis with 95% confidence intervals for the AUC calculated using the DeLong method, and optimal cut-offs were derived using Youden’s index. Paired comparisons of AUCs derived from the same patients were made with the DeLong test. Univariable and multivariable logistic-regression models were constructed for each chemokine and for the three-chemokine panel. A two-sided *p* < 0.05 was considered statistically significant.

Graphics were produced from the author-verified estimates using AI-assisted plotting code (see the Acknowledgments section).

## 3. Results

### 3.1. Patient Characteristics

The median age in the ALD group was 49.0 years (IQR: 41.0–58.0). Forty-four patients were male (69.8%), and 19 were female (30.2%). Healthy controls (*n* = 25) were group-matched for age and sex. CTP class distribution was A in 16 patients (25.4%), B in 28 (44.4%) and C in 19 (30.2%). The short-term (30-day) mortality was 7/63 (11.1%). Characteristics of the study cohort and their inflammatory indices are presented in Table 2 and Table 3.

The following clinical complications of ALD were recorded at admission: ascites in 40/63 (63.5%), oesophageal varices in 33/63 (52.4%) and hepatic encephalopathy in 2/63 (3.2%). ALD-related death occurred in 7/63 (11.1%) patients (1/19 women, 6/44 men; *p* = 0.6643) during the 30-day follow-up period.

### 3.2. Comparison of Serum CC-Chemokine Concentrations: ALD Group Versus Controls

**Figure 1:**
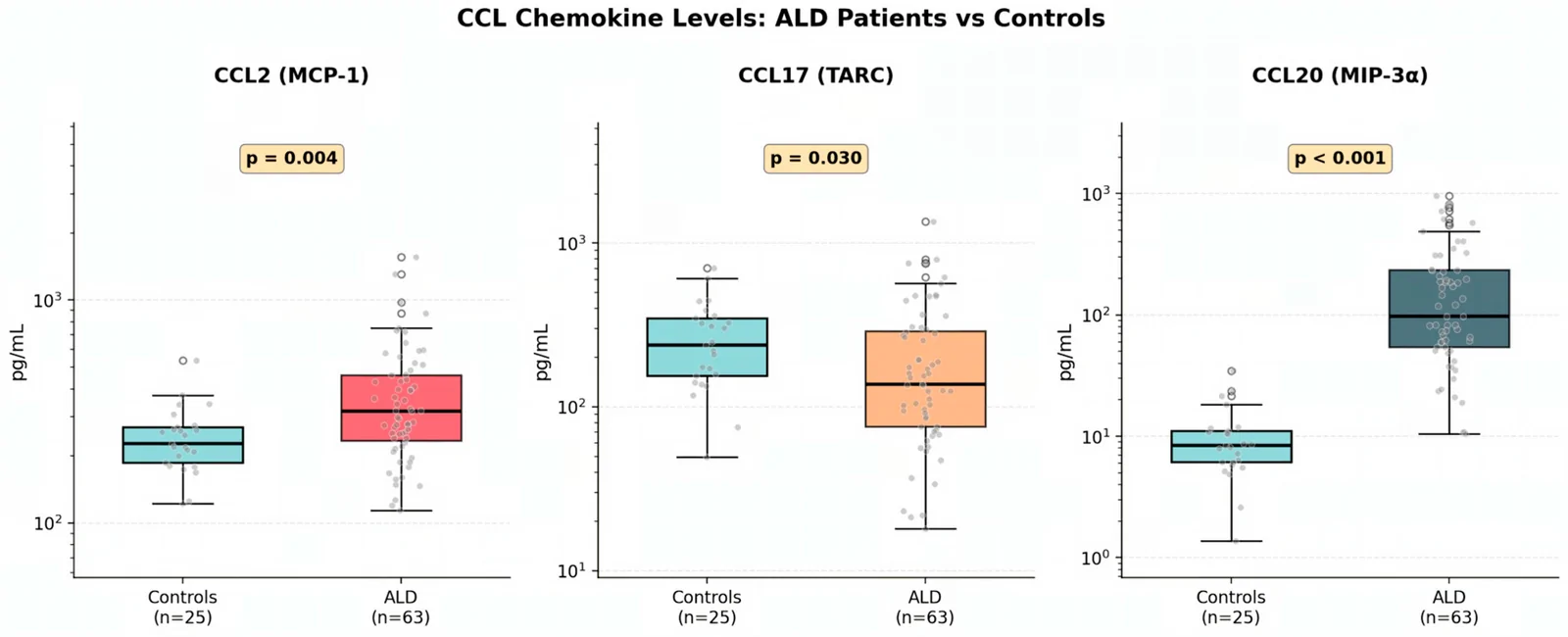
Serum CC-chemokine concentrations in ALD versus controls (log scale; Mann–Whitney U test).

All studied CC-chemokine blood levels differed significantly between ALD patients and controls. MIP-3α/CCL20 showed the largest separation between ALD and controls—an 11-fold rise that translated into a near-perfect effect size (r = 0.96). MCP-1/CCL2 was also significantly higher in ALD patients, whereas TARC/CCL17 went the other way, with values significantly lower in ALD than in controls. The results are presented in Table 4 and Figure 1.

### 3.3. Correlations of the Studied CCLs with Inflammatory Indices

Correlations of CCLs with inflammatory indices in the ALD group are presented in Table 5. MIP-3α/CCL20 correlated strongly with CRP and white blood cell (WBC) counts (except lymphocytes), as well as with NLR and SII. MCP-1/CCL2 presented a weaker positive association only with CRP. TARC/CCL17 showed a positive correlation only with lymphocyte count, but no association with the other inflammatory indices. All associations that were significant at *p* < 0.05 remained significant after Benjamini–Hochberg correction (q < 0.05).

### 3.4. Correlations of the Studied CCLs with Liver Tests and Prognostic Scales

**Figure 2:**
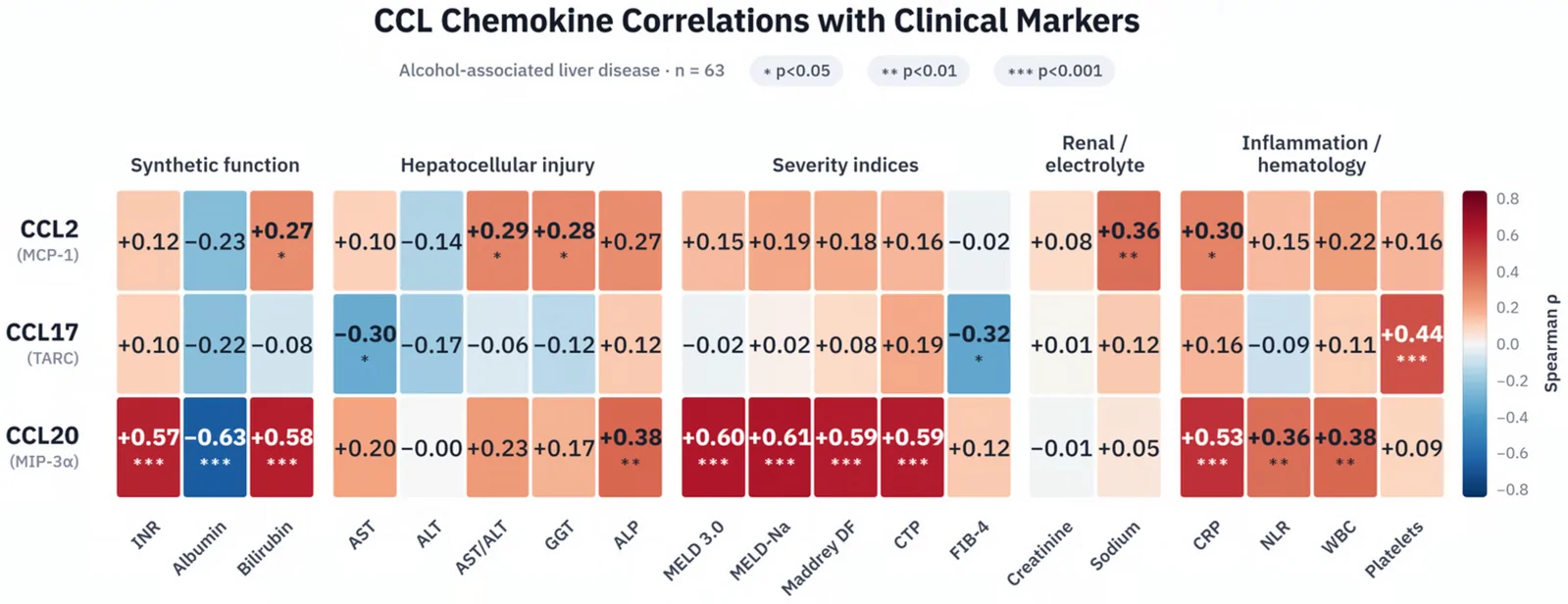
Spearman correlations of serum CC-chemokines (rows) with hepatic biochemical and prognostic parameters (columns) in patients with ALD (*n* = 63). Colour intensity encodes the correlation coefficient; asterisks denote raw two-sided significance.

The relationships of each chemokine with hepatic biochemistry and severity scores are summarised in Figure 2. MIP-3α/CCL20 correlated strongly with three markers of hepatic synthetic function—bilirubin, albumin and INR—the biochemical triad that defines decompensated cirrhosis. It also correlated with alkaline phosphatase but not with the transaminases. MCP-1/CCL2 showed weak associations with bilirubin, the AST/ALT ratio and GGT. TARC/CCL17 showed a weak inverse correlation with AST.

**Figure 3:**
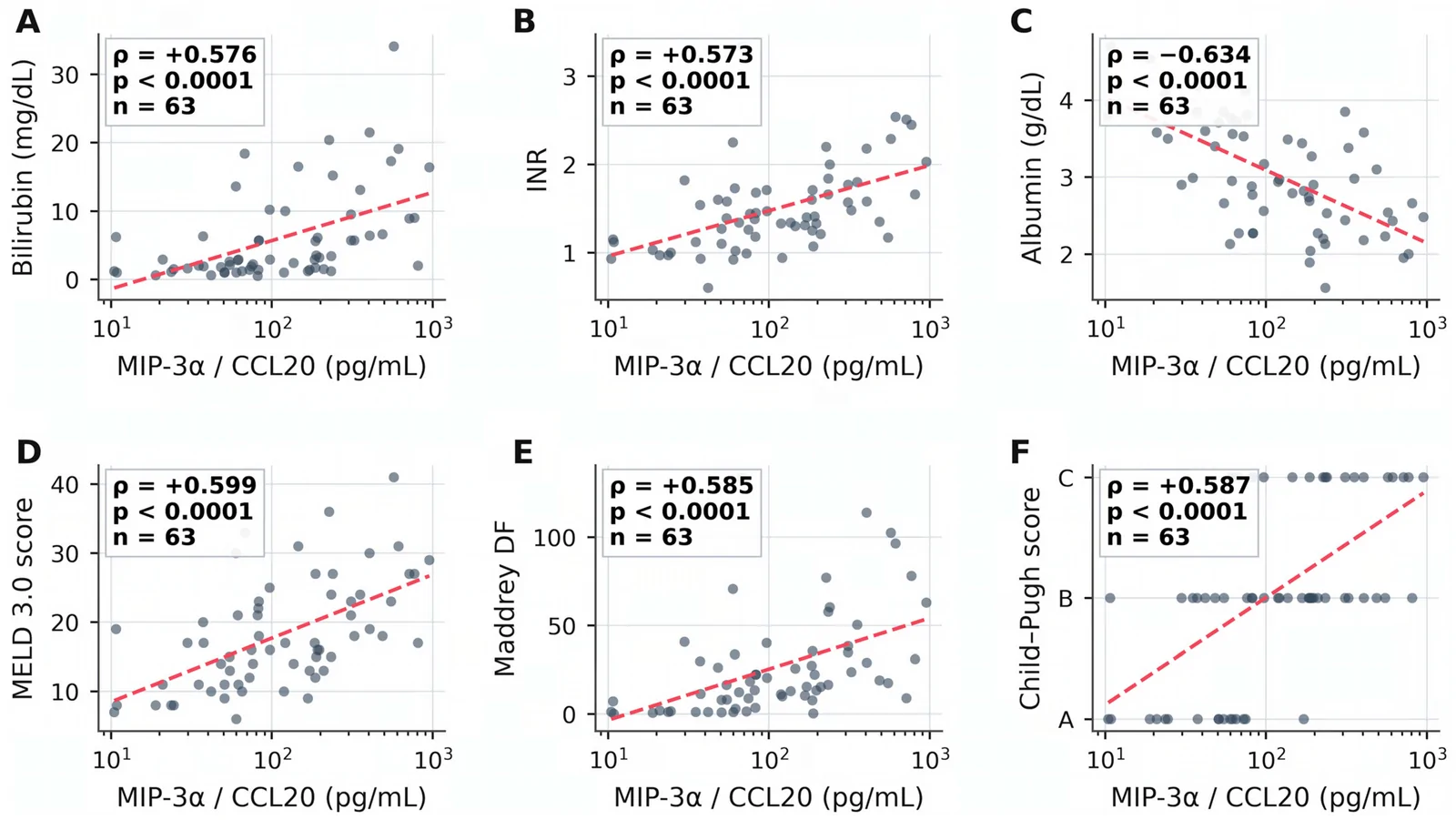
Strongest individual correlations of serum MIP-3α/CCL20 in the ALD cohort. Scatter plots for the six parameters most strongly associated with MIP-3α/CCL20: serum bilirubin (**A**), INR (**B**), albumin (**C**), MELD 3.0 (**D**), modified Maddrey discriminant function (mDF) (**E**) and CTP score (**F**). Each panel reports Spearman ρ and the raw *p*-value.

MIP-3α/CCL20 was the only chemokine that correlated with all four prognostic scales used in ALD (mDF, CTP, MELD-Na and MELD 3.0; each *p* < 0.001). The associations remained significant in partial rank-correlation analysis after adjustment for age and sex (partial ρ = 0.586–0.609, all *p* < 0.001), confirming that MIP-3α/CCL20 reflects disease biology rather than demographic confounding. MCP-1/CCL2 and TARC/CCL17 did not correlate with any prognostic scale. The strongest individual correlations of MIP-3α/CCL20 with hepatic synthetic and prognostic parameters are illustrated in Figure 3.

### 3.5. Comparisons of CC-Motif Chemokine Blood Levels Stratified by CTP Class and MELD Severity Tertiles

**Figure 4:**
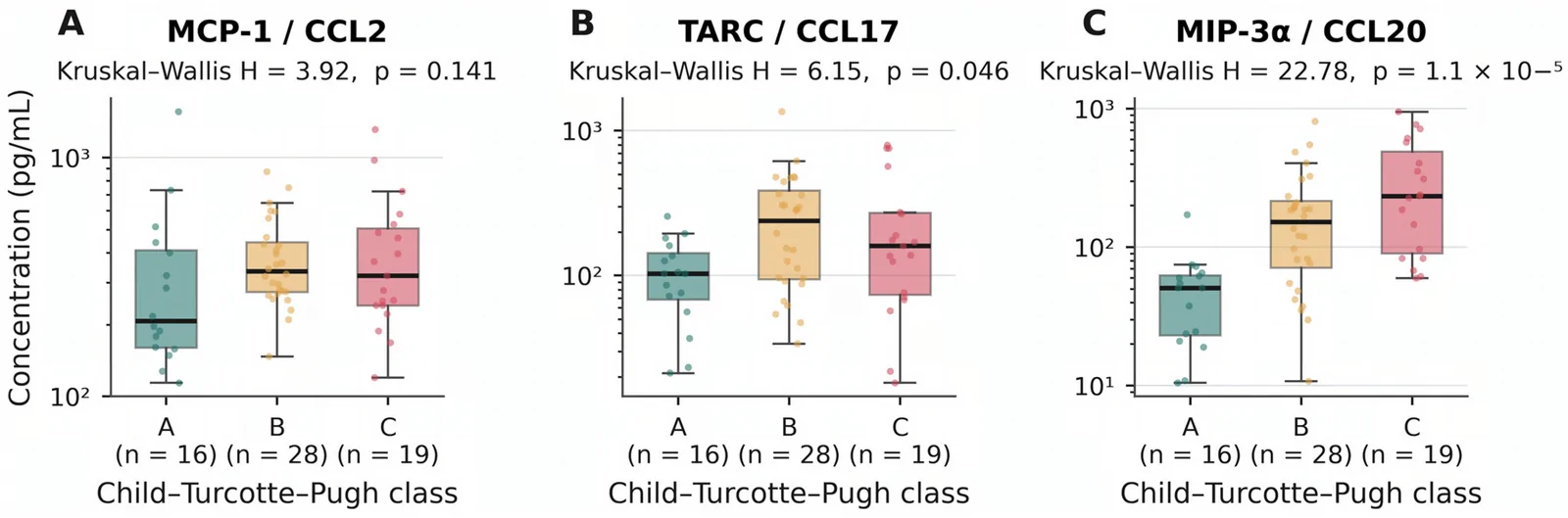
CC-chemokine concentrations stratified by Child–Turcotte–Pugh (CTP) class in patients with ALD (*n* = 63). Each grey dot represents an individual patient.

MIP-3α/CCL20 increased stepwise across CTP classes A, B and C (Table 6, Figure 4). Bonferroni-corrected post-hoc testing showed that CTP class A patients had significantly lower values than both CTP class B and CTP class C, while the difference between class B and class C did not reach statistical significance. TARC/CCL17 also varied across CTP classes, but the pattern was non-monotonic, with the highest values in class B (Table 6; Figure 4, middle panel). MCP-1/CCL2 did not differ between CTP classes.

**Figure 5:**
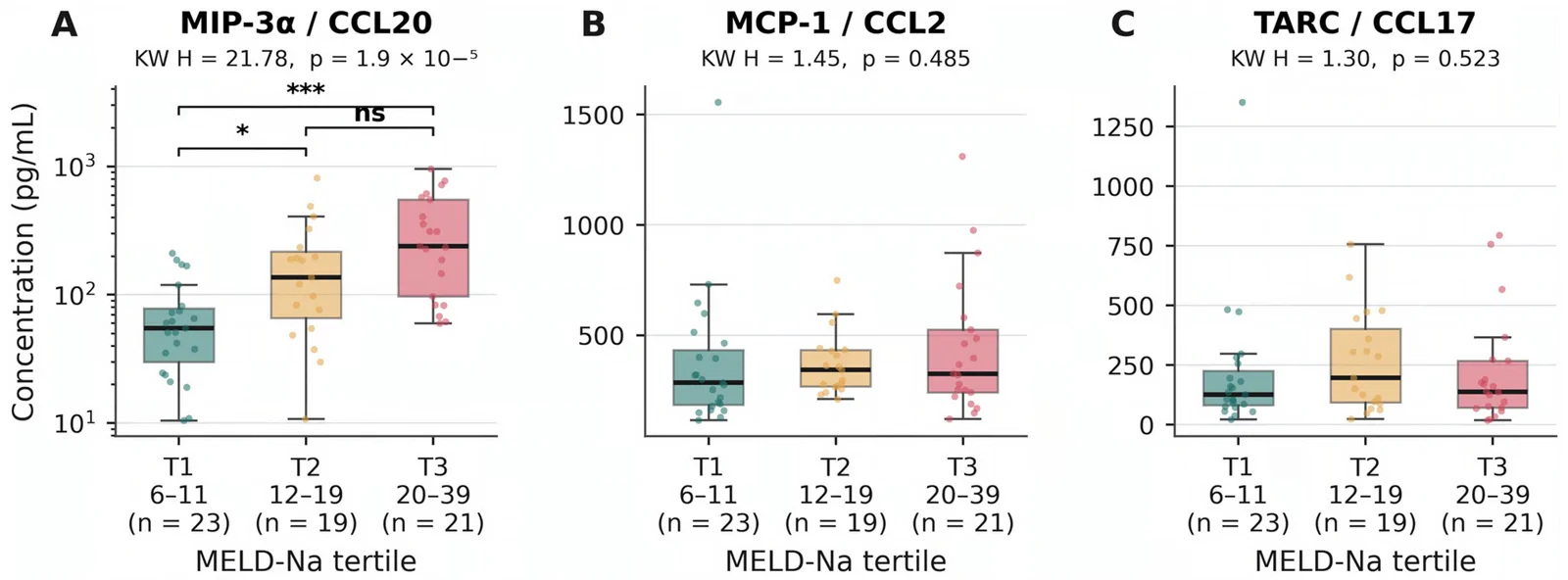
Blood CC-chemokine concentrations across MELD-Na severity tertiles. KW, Kruskal–Wallis test; ns, non-significant. * *p* < 0.05; *** *p* < 0.001.

A similar gradient was observed across MELD-Na severity tertiles for MIP-3α/CCL20. In contrast, neither MCP-1/CCL2 nor TARC/CCL17 differed across tertiles. The results are presented in Figure 5 and Table 7.

The pattern was identical to that for MELD-Na when patients were stratified by MELD 3.0.

### 3.6. Diagnostic and Severity Performance of the Studied CCLs in Patients with ALD

**Figure 6:**
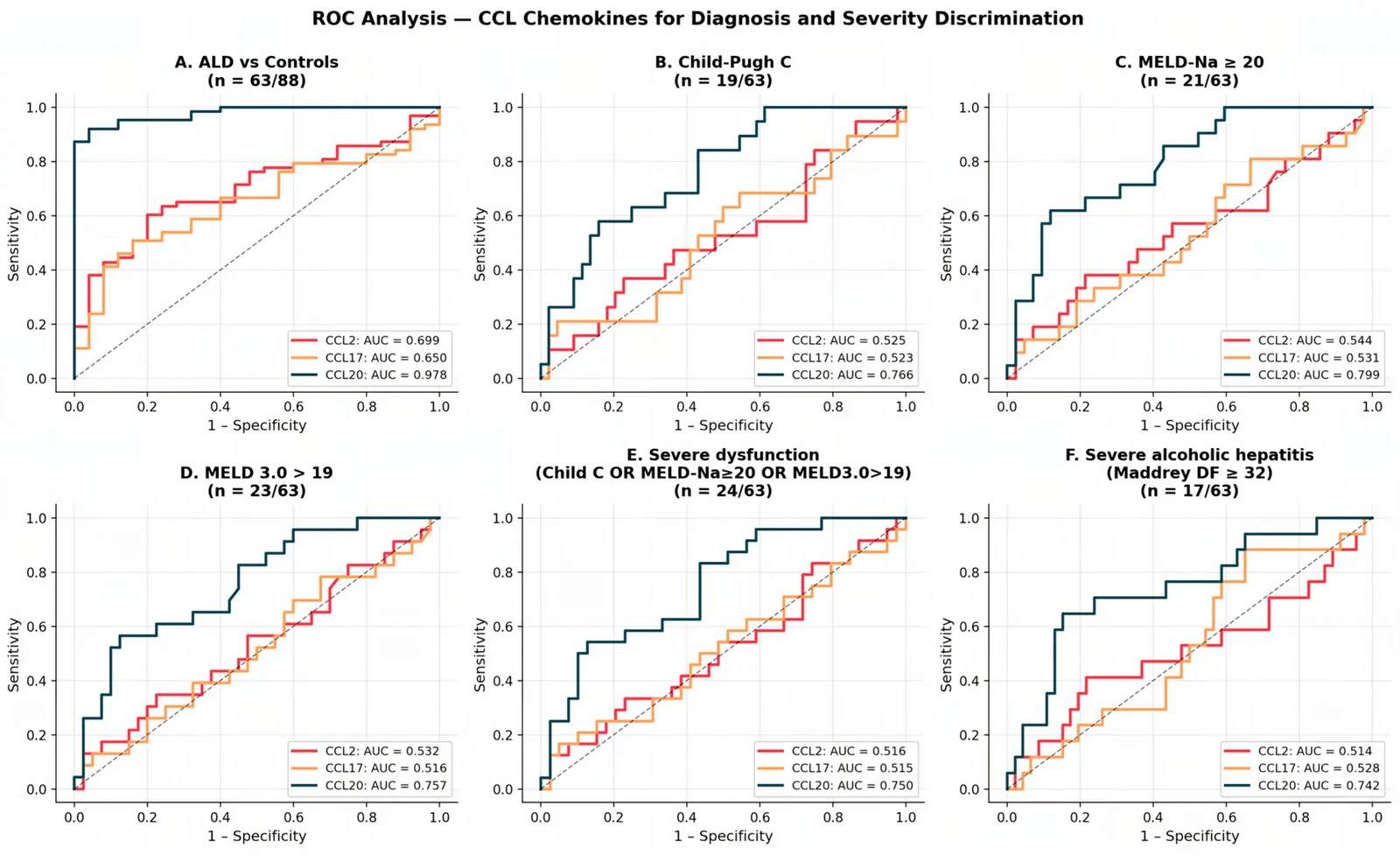
ROC-curve analyses for ALD diagnosis (**A**) and for discrimination of the severity of liver dysfunction (**B**–**F**). AUC, area under the receiver-operating-characteristic curve.

MIP-3α/CCL20 reached an AUC of 0.978 for distinguishing ALD from healthy controls. MCP-1/CCL2 was a fair discriminator (AUC: 0.699), and TARC/CCL17 was a discriminator in the inverse direction (values lower in ALD; AUC: 0.650) (Figure 6). Within the ALD group, MIP-3α/CCL20 was able to identify patients with severe liver dysfunction by each pre-defined criterion separately and the composite (any-criterion) definition, and to identify patients with severe AH, as presented in Table 8 and Figure 6. Youden’s index identified an optimal cut-off of 226.9 pg/mL for all severity endpoints.

Two observations emerged across the severity panels:

(1) MCP-1/CCL2 and TARC/CCL17 performed at or near the chance level (AUC: 0.51–0.55) for every severity definition. Despite elevated MCP-1/CCL2 and reduced TARC/CCL17 in ALD overall, neither chemokine resolved disease severity beyond what would be expected by random ranking.
(2) MIP-3α/CCL20 was the only useful severity discriminator (AUC: 0.74–0.80). A single Youden-optimal cut-off of 226.9 pg/mL recurred across all severity definitions. Nevertheless, this cut-off was derived and applied within the same single cohort and is therefore cohort- and assay-dependent. It requires prospective, multicentre validation before it can be regarded as a clinical threshold.

**Figure 7:**
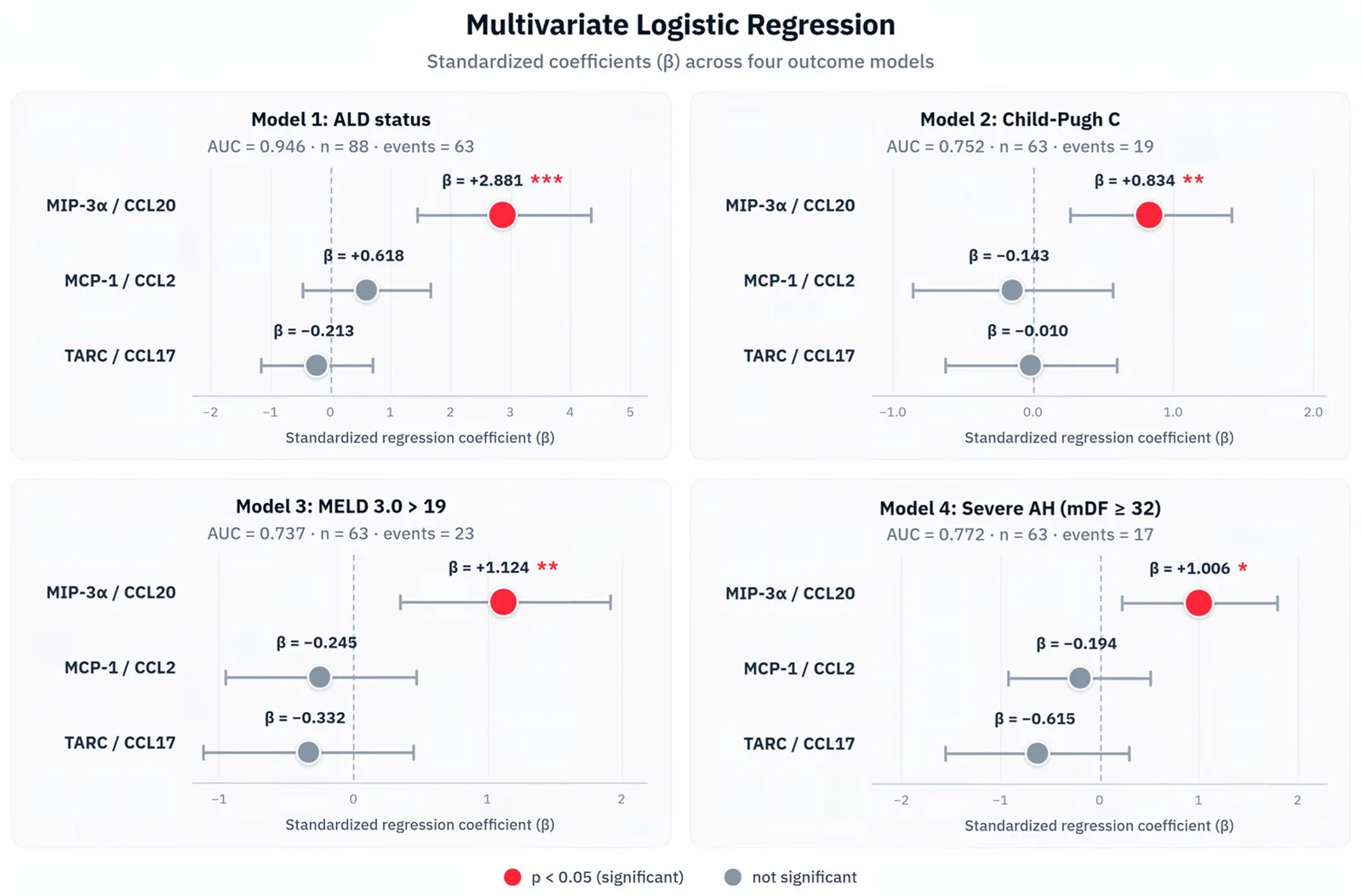
Forest plot of standardised regression coefficients with 95% confidence intervals (CIs) for four multivariable logistic-regression models. Red, statistically significant (*p* < 0.05); grey, not significant. * *p* < 0.05; ** *p* < 0.01; *** *p* < 0.001.

In a multivariable logistic-regression model with all three chemokines entered, MIP-3α/CCL20 was the dominant predictor of ALD status (standardised β = +2.881), with a small contribution from MCP-1/CCL2 (β = +0.618) and an inverse contribution from TARC/CCL17 (β = −0.213). Consequently, the three-chemokine model underperformed MIP-3α/CCL20 alone for ALD-status discrimination (0.946 vs. 0.978). For severe liver dysfunction within the ALD group and for severe AH, MIP-3α/CCL20 was again the only significant predictor in all models; neither TARC/CCL17 nor MCP-1/CCL2 contributed independently. The results are presented in Figure 7.

### 3.7. Relationship of the Studied CC-Chemokines with the FIB-4 Index

FIB-4 is a widely used non-invasive fibrosis score. We compared it directly with the studied CCLs within the ALD cohort. The corresponding correlation coefficients are shown in the severity-indices block of Figure 2. Median FIB-4 was 6.79 (IQR: 3.90–9.86). FIB-4 correlated only weakly with the composite prognostic scales (Spearman ρ = 0.255 for mDF, 0.372 for MELD-Na, 0.343 for MELD 3.0 and 0.323 for CTP; all *p* < 0.05) and was higher in patients with severe liver dysfunction than in those without (7.38 vs. 5.95, *p* = 0.015). MIP-3α/CCL20 did not correlate with FIB-4 (ρ = 0.118, *p* = 0.357), nor did MCP-1/CCL2 (ρ = −0.022, *p* = 0.862). TARC/CCL17 was the only chemokine that correlated with FIB-4, and did so inversely (ρ = −0.317, *p* = 0.011). For discrimination of severe liver dysfunction, MIP-3α/CCL20 yielded numerically higher AUCs than FIB-4 at every endpoint tested (Table 9); for the composite endpoint, the difference did not reach statistical significance on the DeLong test (0.750 vs. 0.684, *p* = 0.49), consistent with the limited number of events.

### 3.8. Associations of CC-Chemokine Blood Levels with ALD Clinical Complications and Outcomes

The next step of our analysis evaluated associations of CC-chemokines with ALD clinical complications and outcome. The results are presented in Table 9 and Table 10.

MIP-3α/CCL20 blood levels were significantly higher in ALD patients with severe liver dysfunction and in those with ascites, and trended higher in patients with oesophageal varices. The other two CC-chemokines did not differ significantly across these subgroups.

The short-term (30-day) mortality was 7/63 (11.1%). Median MIP-3α/CCL20 was higher in non-survivors than in survivors, but the difference did not reach statistical significance. Neither MCP-1/CCL2 nor TARC/CCL17 discriminated between these subgroups. Relative to controls, MCP-1/CCL2 and MIP-3α/CCL20 were higher in both survivors and non-survivors, whereas TARC/CCL17 was lower in both groups, significantly so for non-survivors (*p* = 0.030) and at borderline significance for survivors (*p* = 0.055). Nevertheless, the small number of deaths limits the statistical power of these comparisons and indicates that larger cohorts would be needed to confirm the prognostic accuracy of CCLs for mortality. The results are shown in Table 11.

### 3.9. Sex- and Age-Related Analyses

No significant sex- or age-related differences were detected for any of the three chemokines in the ALD group.

## 4. Discussion

CC-motif chemokines orchestrate immune-cell trafficking, inflammation and fibrogenesis in chronic liver injury. Their dysregulation is established in metabolic dysfunction-associated steatotic liver disease (MASLD), viral hepatitis and autoimmune liver disorders, but data in ALD are limited and rarely accompanied by simultaneous assessment of validated prognostic scores. Pre-clinical studies have linked CCL20, CCL2 and CCL17 to inflammation and fibrosis in cell lines and animal models of ALD [10,18], yet no clinical study to date has profiled them together in a well-characterised ALD cohort. We measured serum MIP-3α/CCL20, MCP-1/CCL2 and TARC/CCL17 in patients with ALD and in healthy controls and correlated the values with conventional inflammatory parameters, routine liver-function tests, prognostic scales and clinical outcome. Our results indicate that the three CC-motif chemokines represent distinct but complementary immunopathological axes. MIP-3α/CCL20 is the most informative biomarker with excellent diagnostic discrimination and correlations with all prognostic scales. MCP-1/CCL2 reflects active hepatic inflammation, and the downregulation of TARC/CCL17 in ALD is, to our knowledge, a novel observation.

### 4.1. MIP-3α/CCL20: A Biomarker with Diagnostic and Prognostic Capability

Serum MIP-3α/CCL20 was markedly raised in patients with ALD compared with in healthy controls. This finding complements the work of Affò et al. [26], who showed that both liver and circulating CCL20 levels in AH significantly exceeded those seen in compensated cirrhotics, in patients with chronic HCV and in healthy volunteers. In that study, hepatic CCL20 expression correlated with the MELD score and with METAVIR fibrosis stage (F4 vs. F1–3), and serum CCL20 was higher in 90-day non-survivors than in survivors. More recent reviews by Feng et al. [38] and Shen et al. [10] have identified CCL20 as one of the most strongly upregulated chemokines in AH, linking it to endotoxaemia, liver fibrosis and short-term mortality. ROC analysis in our cohort yielded an AUC of 0.978 for distinguishing ALD from controls. This represents excellent separation, but the comparison is between sick inpatients and healthy volunteers and is consequently subject to spectrum bias. The figure should therefore be interpreted as a proof-of-principle, case-control result rather than as clinical diagnostic accuracy. Confirmation of its true diagnostic value would require comparison with other chronic liver diseases (e.g., MASLD, viral hepatitis).

Serum MIP-3α/CCL20 correlated positively with all four prognostic scoring systems used in ALD (CTP, MELD-Na, MELD 3.0 and mDF). The associations were independent of age and sex on partial rank-correlation analysis, indicating that MIP-3α/CCL20 reflects disease biology rather than demographic confounding. The findings are consistent with the report of Soliman et al. [39], who showed a correlation between serum CCL20 and the CTP score in cirrhotics, and with that of Affò et al. [33], who reported a correlation between hepatic CCR6 expression and both CCL20 and MELD in patients with AH.

MIP-3α/CCL20 did not correlate with FIB-4 even though it correlated strongly with every composite prognostic score and it discriminated severe liver dysfunction better (i.e., yielded higher AUCs) than FIB-4 at each endpoint tested (Table 9). This dissociation is informative: FIB-4 is weighted towards age, platelet count and transaminases and estimates cumulative fibrotic burden, whereas MIP-3α/CCL20 appears to track the active inflammatory process that drives decompensation. The two markers are therefore complementary rather than interchangeable, and MIP-3α/CCL20 should not be regarded as a surrogate for fibrosis stage.

We observed that MIP-3α/CCL20 increased stepwise across CTP classes A–C and across MELD-Na tertiles. Post-hoc analysis confirmed that CTP class A patients had significantly lower values than CTP class B or class C. Within the ALD group, ROC analysis produced AUCs of 0.766 for CTP class C and 0.799 for MELD-Na ≥ 20. On multivariable logistic regression, MIP-3α/CCL20 was the dominant chemokine predicting both ALD status and severe disease, while MCP-1/CCL2 and TARC/CCL17 did not.

CTP and MELD scores indicate hepatic synthetic and excretory function but do not capture the inflammatory cascade that drives decompensation. MIP-3α/CCL20 may provide a direct molecular biomarker of that cascade. Feng et al. [38] concluded that the inflammatory presentation of AH is heterogeneous, which is probably the reason why uniform anti-inflammatory regimens have failed in different AH patients in clinical trials. Biomarkers tied to specific inflammatory pathways, rather than composite functional scores, may support a more precise treatment approach. Serum MIP-3α/CCL20 could be used as a companion marker in future trials of CCR6 antagonists or anti-CCL20 antibodies in ALD. Should CCR6-directed agents (small-molecule CCR6 antagonists or anti-CCL20 antibodies) advance into ALD trials, serum MIP-3α/CCL20 could plausibly serve two roles: enrichment of trial populations for patients with a demonstrably active CCL20/CCR6 axis and a pharmacodynamic readout of target engagement. Neither role is established by the present data, and both would require prospective, assay-standardised evaluation.

The simultaneous association of MIP-3α/CCL20 with markers of hepatic synthetic function (bilirubin, albumin and INR) and with markers of systemic inflammation (CRP, WBC, neutrophils, NLR and SII) positions this chemokine as a bridging biomarker between the inflammatory cascade and hepatocellular failure. Affò et al. [26] identified macrophages and HSCs as the principal hepatic sources of CCL20, both activated at the interface of inflammation and fibrogenesis. They reported a strong positive correlation between serum CCL20 and LPS levels and showed that silencing CCL20 reduced LPS-induced hepatic injury and pro-inflammatory and pro-fibrogenic gene induction. The high diagnostic discrimination of MIP-3α/CCL20 in our cohort is likely to reflect, in part, gut–liver-axis-dependent CCL20 production driven by LPS exposure—a mechanism uniquely prominent in alcohol-related injury [26,33].

Elevated CCL20 has also been reported across other chronic liver diseases. In MASLD, both Chu et al. [40] and Hanson et al. [41] reported a stepwise increase in CCL20 with steatosis, inflammation and fibrosis, with HSCs identified as a key source. Du et al. [42] reported that the combination of CCL20 and LCN2 reached an AUC of 0.927 for HCC detection. Together with our findings, these data suggest that MIP-3α/CCL20 may represent a near-universal marker of HSC activation and fibrogenesis across chronic liver injury.

### 4.2. MCP-1/CCL2: A Marker of Hepatic Inflammation

MCP-1/CCL2 was moderately raised in ALD versus controls and correlated with CRP, as well as with bilirubin, AST/ALT ratio and gamma-glutamyl transferase. Unlike MIP-3α/CCL20, MCP-1/CCL2 did not correlate with any prognostic scale, did not differ between CTP classes and did not distinguish survivors from non-survivors.

The pattern is consistent with the role of MCP-1/CCL2 as a broad-spectrum monocyte and macrophage chemoattractant. In the clinical series reported by Degré et al. [20], circulating CCL2 was raised across the ALD spectrum and tracked disease severity, with the mDF ≥ 32 subgroup showing higher plasma values than patients with milder AH. Hepatic CCL2 expression correlated with neutrophil infiltration and IL-8 expression, and a trend towards decreased plasma CCL2 was observed after seven days of corticosteroid therapy. Our finding that CCL2 tracks inflammatory markers and selected liver-function parameters but not composite prognostic scores is consistent with the interpretation that it reflects active hepatic inflammation rather than the severity of hepatocellular failure. Queck et al. [23] supported this view by showing that the injured liver is the principal source of systemic MCP-1/CCL2, with portal and hepatic-vein concentrations exceeding systemic levels.

Pre-clinical studies have established the mechanistic role of the CCL2/CCR2 axis in ALD. Mandrekar et al. [22] observed that mice lacking MCP-1 were resistant to the hepatic consequences of chronic alcohol exposure, as both lipid accumulation and cytokine induction were lower than in wild-type controls. CCR2-knockout mice were not protected, suggesting that some effects of MCP-1 in ALD are CCR2-independent and may include direct interference with PPARα-mediated lipid metabolism in hepatocytes. Such complexity may explain why the elevation of MCP-1/CCL2 in our cohort was moderate rather than marked: unlike CCL20, which is induced directly by LPS through TLR4, CCL2 induction involves multimodal signalling. Sasaki et al. [43] identified an additional regulatory mechanism of CCL2 in the liver of ALD patients: NADPH oxidase 4 (NOX4), a source of ROS, was upregulated, localised to activated HSCs, and associated with prolonged half-life of CCR2 and CCL2 transcripts, sustaining CCR2/CCL2 signalling at the post-transcriptional level.

The therapeutic relevance of CCL2/CCR2 signalling was illustrated by Ambade et al. [24], who showed that the dual CCR2/CCR5 antagonist cenicriviroc prevented and reversed alcohol-induced liver damage, steatosis and inflammation in mice and lowered the accumulation of pro-inflammatory Ly6C^high^ macrophages. The phase III AURORA trial of cenicriviroc in non-alcoholic steatohepatitis subsequently failed to meet its fibrosis endpoint, illustrating the difficulty of translating single chemokine-receptor blockade into clinical practice—a difficulty often attributed to redundancy in chemokine-signalling networks [44]. Baeck et al. [45,46] found that blocking CCL2 decreased hepatic macrophage numbers, attenuated steatohepatitis and allowed more rapid fibrosis reversal in mouse models. The blockade shifted the intrahepatic macrophage compartment away from the inflammatory Ly-6C^high^ towards reparative Ly-6C^low^ cells. MCP-1/CCL2 therefore contributes to ALD pathogenesis in a context-dependent manner.

### 4.3. TARC/CCL17: A Paradoxical Decrease in ALD

The novel finding of this study was the significantly lower serum TARC/CCL17 levels in patients with ALD than in healthy controls. Nevertheless, because these data are associative and derived from a single cohort, the reduced TARC/CCL17 should be regarded as a hypothesis requiring dedicated mechanistic testing in pre-clinical ALD models rather than as an established mechanism. CCL17 correlated positively with lymphocyte and platelet counts and inversely with AST. It did not correlate with any prognostic scale and did not separate survivors from non-survivors. However, it differed across CTP classes, but in a non-monotonic pattern. To our knowledge, this is the first clinical report on serum CCL17/TARC in ALD. An extensive literature search returned no previous studies of CCL17/TARC concentrations in ALD, AH or alcohol-associated cirrhosis.

CCL17/TARC is a ligand for CCR4, expressed predominantly on Th2 lymphocytes and Tregs. Oo et al. [29] showed that healthy liver tissue does not express either CCL17 or CCL22, whereas both chemokines appear in chronic hepatitis and are produced by DCs that populate inflammatory foci. Hepatic CCR4+ Tregs in their model responded to CCL17/CCL22 secreted by intrahepatic DCs. Zhang et al. [32] showed that intrahepatic CCL17 mRNA was elevated in chronic hepatitis B (CHB) and promoted recruitment of IL-17-secreting T cells. In contrast to our findings in ALD, they also observed that CCL20 expression was lower in CHB. The elevated CCL17 and decreased CCL20 in viral hepatitis suggests that the immunological microenvironment in ALD differs from that in viral liver disease.

Several mechanisms may account for the lower CCL17 in our cohort. First, ALD shows predominant Th1/Th17 polarisation, as documented by Lemmers et al. [47] and reviewed by Feng et al. [38] and Shen et al. [10]. Chronic alcohol exposure expands IL-17-producing T cells and reduces production of Th2-associated chemokines. Second, chronic alcohol consumption impairs dendritic cell development and function [10], which would in turn reduce hepatic CCL17 production by intrahepatic dendritic cells [29]. Third, the inverse correlation of CCL17 with FIB-4 (ρ = −0.317, *p* = 0.011; Figure 2, Section 3.7) suggests a decline as fibrosis advances, which may reflect loss of intrahepatic dendritic cell populations as liver architecture is disrupted by fibrotic remodelling and nodule formation. The liver-transplant series of Meng et al. [31] revealed that serum TARC/CCL17 was highest when rejection was mildest and lowest when rejection was most advanced, which the authors interpreted as collapse of a regulatory response under severe injury.

The clinical implication is that the immunoregulatory arm of the hepatic immune response may be functionally diminished in advanced ALD. CCL17-recruited CCR4+ Tregs normally restrain hepatic inflammation [29], so reduced CCL17-driven Treg recruitment may contribute to the unopposed Th1/Th17-driven inflammatory milieu that characterises progressive ALD and is consistent with the previously described Th17/Treg imbalance in ALD [18,47,48]. In HCC, by contrast, Zhou et al. [34] showed that CCL17 was among the most highly expressed chemokines in tumour-associated neutrophils, recruiting CCR4+ Tregs and correlating with poor prognosis. CCL17 appears to behave differently across liver disease etiologies: upregulated in HCC and viral hepatitis, and downregulated in ALD. This complexity suggests that further mechanistic investigation is necessary before any therapeutic intervention targeting the CCL17 axis in ALD is considered. Nevertheless, because these data are associative and derived from a single cohort, the reduced TARC/CCL17 should be regarded as a hypothesis requiring dedicated mechanistic testing in pre-clinical ALD models rather than as an established mechanism.

### 4.4. Differential Chemokine Profiles: A Multi-Axis Model of ALD Immunopathology

Simultaneous measurement of three CC-motif chemokines in a single ALD cohort reveals distinct, complementary axes of disease biology. MIP-3α/CCL20 marks disease severity and hepatocellular failure, possibly through CCR6-dependent recruitment of Th17 cells and DCs, which drive inflammation and fibrogenesis. MCP-1/CCL2 captures hepatic inflammatory activity through CCR2-dependent monocyte and macrophage recruitment but does not stratify severity. TARC/CCL17 reflects the CCR4-dependent Th2/Treg pathway. Its decrease in ALD suggests an immunoregulatory imbalance that may lead to Th1/Th17-driven hepatic injury. The framework extends the model described by Marra and Tacke [18] and updated by Cao et al. [49], who emphasised the interplay between pro-inflammatory and regulatory chemokine axes in liver disease.

These findings complement our companion study of CXC-motif chemokines (CXCL9, CXCL10 and CXCL16) in the same patient cohort [35], in which CXCL9 and CXCL10 emerged as the strongest predictors of ALD severity and short-term mortality. Together, the two studies provide chemokine characteristics in ALD patients which combine the CC family (innate monocyte/macrophage recruitment via CCL2; Th17 recruitment via CCL20; Treg/Th2 modulation via CCL17) and the CXC family (Th1/NK-cell recruitment via CXCL9/CXCL10; NKT-cell recruitment via CXCL16). As Feng et al. [38] noted, the crosstalk between immune cells and liver non-parenchymal cells (macrophages, neutrophils, HSCs and LSECs) generates complex cytokine and chemokine signalling. Our CC-motif chemokine data, together with the previous CXCL data, add new evidence on those intrahepatic interactions that can be confirmed in the peripheral blood of ALD patients.

### 4.5. Chemokines and Inflammatory Haematological Indices

MIP-3α/CCL20 correlated positively with NLR, SII and inflammatory cell counts (WBC, neutrophils). Shen et al. [10] and Feng et al. [38] noted that both circulating and hepatic neutrophil counts are elevated in AH and that NLR correlates with mortality. Forrest et al. [50], in their analysis of 789 STOPAH participants, observed that corticosteroids conferred a survival benefit only in the intermediate-NLR band (5–8) and that a combined Lille–NLR model reached an AUC of 0.889, outperforming either score alone. Our data suggest that MIP-3α/CCL20 may be one of the molecular mediators behind the prognostic value of NLR, since MIP-3α/CCL20 drives Th17-associated neutrophilic inflammation. This implies that a combined score integrating conventional scales with chemokine measurements may improve risk stratification.

TARC/CCL17 showed the opposite pattern, with a positive correlation with lymphocyte count consistent with its role in recruiting lymphocyte subsets (Th2 cells, Tregs) that maintain immune homeostasis [30]. Our data support a Th17/Treg imbalance in ALD, in which MIP-3α/CCL20 drives the inflammatory arm and TARC/CCL17 reflects the failing regulatory arm. MCP-1/CCL2 sits between the two, with intermediate correlations with CRP, as expected for a general inflammatory mediator rather than a driver of a specific immune cell axis.

### 4.6. Mortality Analysis

MIP-3α/CCL20 was higher in non-survivors than in survivors, but the difference did not reach statistical significance; MCP-1/CCL2 and TARC/CCL17 did not separate the two groups, either. The most likely explanation is the small number of non-survivors in our cohort, which markedly limits statistical power. With only seven deaths, this analysis is strictly exploratory and underpowered and should not be interpreted as definitive prognostic evidence. Affò et al. [26] reported that patients who died within 90 days had hepatic CCL20 expression about three-fold higher than survivors and serum levels nearly twice as high. Future multicentre studies are therefore warranted to clarify this point. Adequately powered, multicentre cohorts with longitudinal sampling are required before any short-term prognostic role for these chemokines can be claimed.

### 4.7. Limitations and Future Directions

Several limitations should be acknowledged. First, this was a single-centre study with a moderate sample size, and the mortality analysis in particular was underpowered (only 7 non-survivors); a further limitation is the use of healthy volunteers as the only comparator. Second, because the diagnostic comparison used healthy volunteers rather than disease controls, the ALD-versus-control AUC is affected by spectrum bias, and the discrimination of these chemokines against other chronic liver diseases is required. Third, biomarker sampling was cross-sectional, so we cannot determine whether chemokine changes precede or follow clinical decompensation. Fourth, liver biopsy was not performed, so correlation with histopathological features (inflammation grade, steatohepatitis severity and METAVIR fibrosis stage) was not possible, and intrahepatic chemokine expression could not be measured. Fifth, although strict inclusion and exclusion criteria were applied, residual confounding cannot be entirely ruled out. In particular, the intensity and recency of alcohol intake, nutritional status and subclinical infection may each influence circulating chemokine concentrations independently of liver-disease severity.

Future multicentre studies with larger cohorts and longer follow-up are needed to validate our findings and to establish clinically useful chemokine cut-offs. Longitudinal assessment of CC-chemokine dynamics in relation to abstinence, relapse and treatment (including corticosteroid therapy) will clarify whether changes in MIP-3α/CCL20 precede clinical decompensation and whether the chemokine can predict disease trajectory. From a translational perspective, the CCL20/CCR6 axis is an attractive treatment target: CCR6 antagonists could attenuate Th17-mediated hepatic injury, and serial MIP-3α/CCL20 measurements could serve as pharmacodynamic markers. Examining these CC-chemokines across other chronic liver disorders (MASLD, viral hepatitis and autoimmune hepatitis) would clarify the ALD-specificity of our findings. The paradoxical decrease of CCL17 blood concentrations deserves dedicated mechanistic investigation in pre-clinical ALD models.

## 5. Conclusions

The present work constitutes an exploratory biomarker analysis, indicating that MIP-3α/CCL20 may serve as an indicator of disease severity in patients with ALD. These findings do not yet establish the chemokine as a diagnostic biomarker ready for clinical use, nor as a companion biomarker for CCR6-targeted therapies. Further confirmation in an independent cohort is necessary, along with longitudinal sampling, to establish the marker’s temporal dynamics and prognostic value. Prospective validation of defined thresholds in multicentre trials is required before any diagnostic or treatment-stratification role can be claimed. MIP-3α/CCL20 did not track the FIB-4 index, suggesting that it reflects inflammatory decompensation rather than cumulative fibrotic burden and that the two markers are complementary. The short-term mortality analysis, based on seven events, was strictly exploratory and underpowered. The novel finding of reduced TARC/CCL17 in ALD points to a separate immunoregulatory failure and warrants further mechanistic study in pre-clinical models.

## Figures and Tables

**Table 1:** Prevalence of severe liver dysfunction and severe alcohol-associated hepatitis in the studied ALD cohort (*n* = 63).

Criterion ^1^	*n* (%)
CTP class C	19 (30.2)
MELD-Na ≥ 20	21 (33.3)
MELD 3.0 > 19	23 (36.5)
Any severe dysfunction	24 (38.1)
Severe AH (mDF ≥ 32)	17 (27.0)

^1^ AH, alcohol-associated hepatitis; CTP, Child–Turcotte–Pugh; mDF, modified Maddrey discriminant function; MELD, Model for End-Stage Liver Disease.

**Table 2:** Study cohort characteristics.

Variable ^1^	ALD (*n* = 63)	Controls (*n* = 25)	*p*
Age (years),	49 (41–58)	45 (33–50)	0.080
Male/female (*n*)	44/19	16/9	0.600
AST (U/L)	120 (81–171)	32 (30–35)	<0.001
ALT (U/L)	46.5 (30–84)	31 (25–36)	0.001
AST/ALT ratio	2.23 (1.58–3.19)	1.06 (0.92–1.16)	<0.001
Bilirubin (mg/dL)	2.9 (1.45–7.75)	0.7 (0.6–0.9)	<0.001
INR	1.40 (1.16–1.71)	0.90 (0.80–1.00)	<0.001
Albumin (g/dL)	2.95 (2.46–3.59)	3.85 (3.70–4.00)	<0.001
Creatinine (mg/dL)	0.80 (0.70–1.00)	0.60 (0.60–0.70)	<0.001
CTP class (A/B/C), *n*	16/28/19	—	—
MELD-Na	15 (10–22)	—	—
MELD 3.0	17 (12–23)	—	—
Maddrey mDF	16.5 (8.5–35)	—	—
FIB-4	6.79 (3.90–9.86)	—	—

^1^ Values are medians (Q1–Q3) unless otherwise stated. ALD, alcohol-associated liver disease; ALT, alanine aminotransferase; AST, aspartate aminotransferase; CTP, Child–Turcotte–Pugh; FIB-4, Fibrosis-4 index; INR, international normalised ratio; mDF, modified Maddrey discriminant function; MELD, Model for End-Stage Liver Disease; Q, quartile. *p*-values are from the Mann–Whitney U test (continuous) or χ^2^ test (sex).

**Table 3:** Inflammatory indices in patients with ALD and controls.

Variable ^1^	ALD (*n* = 63)	Controls (*n* = 25)	*p*
CRP (mg/L)	22.0 (5.9–48.1)	1.97 (1.22–2.33)	<0.001
WBC (×10^9^/L)	7.18 (4.83–9.65)	5.23 (4.87–6.12)	0.002
Neutrophils (×10^9^/L)	4.86 (3.15–8.08)	3.21 (2.98–3.80)	0.001
Lymphocytes (×10^9^/L)	1.16 (0.85–1.46)	1.57 (1.33–1.66)	<0.001
Platelets (×10^9^/L)	131 (95.5–171.5)	301 (287–320)	<0.001
NLR	4.09 (2.48–7.20)	2.18 (2.00–2.31)	<0.001
SII	501.8 (279.1–1126.7)	651.0 (596.7–708.5)	0.157

^1^ Values are medians (IQR). ALD, alcohol-associated liver disease; CRP, C-reactive protein; IQR, interquartile range (25th–75th percentile); NLR, neutrophil-to-lymphocyte ratio; SII, systemic immune-inflammation index; WBC, white blood cell. *p*-values are from the Mann–Whitney U test.

**Table 4:** Serum chemokine concentrations in patients with ALD and healthy controls.

Chemokine (pg/mL) ^1^	Controls (*n* = 25) Median (IQR)	ALD (*n* = 63) Median (IQR)	Mann– Whitney U	*p*-Value	Effect Size (r)
TARC/CCL17	237.54 (155.18–347.21)	137.93 (76.07–291.55)	552.0	0.030 *	0.30
MCP-1/CCL2	227.89 (186.38–268.90)	318.22 (235.14–462.41)	1101.0	0.004 **	0.40
MIP-3α/CCL20	8.41 (6.13–11.11)	97.24 (54.65–233.73)	1540.0	<0.001 ***	0.96

^1^ IQR, interquartile range; r, Mann–Whitney rank-biserial correlation. * *p* < 0.05; ** *p* < 0.01; *** *p* < 0.001.

**Table 5:** Spearman correlations of serum CC-motif chemokines with inflammatory markers in patients with ALD (*n* = 63).

Chemokine ^1^	CRP	WBC	NEU	LYMF	NLR	SII
TARC/CCL17	+0.16	+0.11	+0.03	+0.34 **	−0.09	+0.19
MCP-1/CCL2	+0.30 *	+0.22	+0.24	+0.14	+0.15	+0.22
MIP-3α/CCL20	+0.53 ***	+0.38 **	+0.39 **	+0.01	+0.36 **	+0.33 **

^1^ Values are Spearman’s rank-correlation coefficients (ρ). CRP, C-reactive protein; LYMF, lymphocytes; NEU, neutrophils; NLR, neutrophil-to-lymphocyte ratio; SII, systemic immune-inflammation index; WBC, white blood cell count. * *p* < 0.05; ** *p* < 0.01; *** *p* < 0.001 (raw, two-sided). Benjamini–Hochberg-adjusted q-values for the significant associations: MIP-3α/CCL20 with CRP q = 0.0002, WBC q = 0.013, NEU q = 0.013, NLR q = 0.015, SII q = 0.028; TARC/CCL17 with LYMF q = 0.025; MCP-1/CCL2 with CRP q = 0.044.

**Table 6:** Serum CC-chemokine concentrations stratified by Child–Turcotte–Pugh class.

Chemokine (pg/mL) ^1^	CTP A (*n* = 16) Median (IQR)	CTP B (*n* = 28) Median (IQR)	CTP C (*n* = 19) Median (IQR)	KW *p*	ε^2^
TARC/CCL17	103.00 (68–143)	238.51 (94–386)	160.18 (74–270)	0.046 *	0.069
MCP-1/CCL2	206.44 (160–410)	333.90 (273–441)	319.55 (240–505)	0.141	0.032
MIP-3α/CCL20	50.76 (23–63)	151.97 (71–216)	233.97 (90–489)	<0.001 ***	0.346

^1^ CTP, Child–Turcotte–Pugh; IQR, interquartile range; KW, Kruskal–Wallis test. ε^2^, epsilon-squared effect size (0.01 = small; 0.06 = medium; 0.14 = large). * *p* < 0.05; *** *p* < 0.001.

**Table 7:** Serum CC-motif chemokine concentrations stratified by MELD-Na severity tertiles.

Chemokine (pg/mL) ^1^	T1: 6–11 (*n* = 23) Median (IQR)	T2: 12–19 (*n* = 19) Median (IQR)	T3: 20–39 (*n* = 21) Median (IQR)	KW *p*	ε^2^
MIP-3α/CCL20	54.8 (29.8–78.1)	136.5 (65.4–215.3)	239.1 (96.8–550.5)	<0.0001 ***	0.330
MCP-1/CCL2	284.4 (183.2–431.5)	342.4 (267.3–431.4)	325.4 (240.5–525.0)	0.485	<0.01
TARC/CCL17	126.3 (80.9–225.4)	195.8 (91.8–402.0)	137.9 (70.9–267.4)	0.523	<0.01

^1^ IQR, interquartile range; KW, Kruskal–Wallis test; MELD, Model for End-Stage Liver Disease; T, tertile; ε^2^, epsilon-squared effect size. *** *p* < 0.001.

**Table 8:** Accuracy of MIP-3α/CCL20 for discrimination of ALD status and severity of liver dysfunction.

ALD Status/Outcome ^1^	AUC	95% CI	Cut-Off (Youden)	Sens %	Spec %
ALD vs. control	0.978	0.951–0.996	23.7 pg/mL	92.1	96.0
Child–Pugh C	0.766	0.634–0.872	226.9 pg/mL	57.9	84.1
MELD-Na ≥ 20	0.799	0.683–0.901	226.9 pg/mL	61.9	88.1
MELD 3.0 > 19	0.757	0.638–0.867	226.9 pg/mL	56.5	87.5
Severe liver dysfunction (any criterion)	0.750	0.631–0.860	226.9 pg/mL	54.2	87.2
Severe AH (mDF ≥ 32)	0.742	0.598–0.869	226.9 pg/mL	64.7	84.8

^1^ AH, alcohol-associated hepatitis; ALD, alcohol-associated liver disease; AUC, area under the receiver-operating-characteristic curve; CI, confidence interval; MELD, Model for End-Stage Liver Disease; mDF, modified Maddrey discriminant function; Sens, sensitivity; Spec, specificity.

**Table 9:** Discriminative performance of MIP-3α/CCL20 compared with the FIB-4 index for severe liver dysfunction in patients with ALD (*n* = 63).

Endpoint ^1^	AUC MIP-3α/CCL20	AUC FIB-4
Severe liver dysfunction (any criterion)	0.750	0.684
Child–Pugh C	0.766	0.663
MELD-Na ≥ 20	0.799	0.689
MELD 3.0 > 19	0.757	0.682
Severe AH (mDF ≥ 32)	0.742	0.669

^1^ AH, alcohol-associated hepatitis; AUC, area under the receiver-operating-characteristic curve; FIB-4, Fibrosis-4 index; MELD, Model for End-Stage Liver Disease; mDF, modified Maddrey discriminant function. All AUCs were computed within the ALD cohort. DeLong test for the composite endpoint: *p* = 0.49.

**Table 10:** CC-chemokine concentrations in ALD patients stratified by clinical complications.

Complication (Present/Total) ^1^	MCP-1/CCL2 Present vs. Absent (*p*)	TARC/CCL17 Present vs. Absent (*p*)	MIP-3α/CCL20 Present vs. Absent (*p*)
Severe liver dysfunction (any criterion) (24/63)	318.88 vs. 298.89 (0.837)	149.05 vs. 136.56 (0.848)	230.45 vs. 72.53 (<0.001)
Ascites (40/63)	343.56 vs. 276.90 (0.059)	172.85 vs. 125.31 (0.177)	186.51 vs. 50.77 (<0.001)
Oesophageal varices (33/63)	319.55 vs. 306.48 (0.842)	160.70 vs. 131.43 (0.596)	145.86 vs. 73.58 (0.064)

^1^ Severe liver dysfunction (any criterion) was defined as CTP class C or MELD-Na ≥ 20 or MELD 3.0 > 19. Ascites and oesophageal varices were coded as binary complications from the clinical record. Values are medians (pg/mL) in patients with versus without each complication; *p*-values are from the two-sided Mann–Whitney U test.

**Table 11:** CC-chemokine concentrations by survival status in the ALD cohort, with control-group reference.

Chemokine (pg/mL) ^1^	ALD Survivors (a) *n* = 56	ALD Non-Survivors (b) *n* = 7	Controls (c) *n* = 25	*p* a vs. b	*p* a vs. c	*p* b vs. c
MCP-1/CCL2	308.55 (226.51–446.05)	325.41 (245.70–777.53)	227.89 (186.38–268.90)	0.406	0.007 **	0.030 *
TARC/CCL17	152.47 (87.03–298.97)	70.89 (62.44–135.44)	237.54 (155.18–347.21)	0.162	0.055	0.030 *
MIP-3α/CCL20	97.00 (50.76–215.83)	226.93 (82.67–488.79)	8.41 (6.13–11.11)	0.106	<0.001 ***	<0.001 ***

^1^ Values are medians (IQR) in pg/mL. *p*-values are from the two-sided Mann–Whitney U test. * *p* < 0.05; ** *p* < 0.01; *** *p* < 0.001.

## Data Availability

The data presented in this study are available on reasonable request from the corresponding author. The data are not publicly available due to ethical and privacy restrictions.

## Abbreviations

The following abbreviations are used in this manuscript:

ACLF	acute-on-chronic liver failure
AH	alcohol-associated hepatitis
ALD	alcohol-associated liver disease
ALP	alkaline phosphatase
ALT	alanine aminotransferase
AST	aspartate aminotransferase
ATP	adenosine triphosphate
AUC	area under the receiver-operating-characteristic curve
AUDIT-C	Alcohol Use Disorders Identification Test–Consumption
BH	Benjamini–Hochberg (procedure)
CBC	complete blood count
CCL2	C-C motif chemokine ligand 2
CCL17	C-C motif chemokine ligand 17
CCL20	C-C motif chemokine ligand 20
CCL22	C-C motif chemokine ligand 22
CCR2	C-C chemokine receptor type 2
CCR4	C-C chemokine receptor type 4
CCR6	C-C chemokine receptor type 6
CD14	cluster of differentiation 14
CI	confidence interval
COVID-19	coronavirus disease 2019
CRP	C-reactive protein
CTP	Child–Turcotte–Pugh (score)
CXCL9	C-X-C motif chemokine ligand 9
CXCL10	C-X-C motif chemokine ligand 10
CXCL16	C-X-C motif chemokine ligand 16
DAMP	damage-associated molecular pattern
DC	dendritic cell
ELISA	enzyme-linked immunosorbent assay
FIB-4	Fibrosis-4 index
Foxp3	Forkhead box P3
GGT	gamma-glutamyl transferase
HCC	hepatocellular carcinoma
HCV	hepatitis C virus
HIV	human immunodeficiency virus
HMGB1	high-mobility group box 1
HSC	hepatic stellate cell
IL-8	interleukin-8
IL-17	interleukin-17
INR	international normalised ratio
IQR	interquartile range
KW	Kruskal–Wallis (test)
LCN2	lipocalin-2
LPS	lipopolysaccharide
LSEC	liver sinusoidal endothelial cell
Ly-6C	lymphocyte antigen 6C
LYMF	lymphocyte count
MASLD	metabolic dysfunction-associated steatotic liver disease
MCP-1	monocyte chemoattractant protein-1
mDF	modified Maddrey discriminant function
MELD	Model for End-Stage Liver Disease
MELD 3.0	Model for End-Stage Liver Disease 3.0
MELD-Na	Model for End-Stage Liver Disease–Sodium
METAVIR	meta-analysis of histological data in viral hepatitis (fibrosis staging system)
MIP-3α	macrophage inflammatory protein-3 alpha
NADPH	nicotinamide adenine dinucleotide phosphate
NAFLD	non-alcoholic fatty liver disease
NEU	neutrophil count
NF-κB	nuclear factor kappa B
NKT	natural killer T (cell)
NLR	neutrophil-to-lymphocyte ratio
NLRP3	NLR family pyrin domain containing 3
NOX4	NADPH oxidase 4
PAMP	pathogen-associated molecular pattern
PMN	polymorphonuclear
ROC	receiver operating characteristic
ROS	reactive oxygen species
SAM	scar-associated macrophage
SBP	spontaneous bacterial peritonitis
SII	systemic immune-inflammation index
STOPAH	steroids or pentoxifylline for alcoholic hepatitis (trial)
TARC	thymus and activation-regulated chemokine
Th1	T helper 1 (cell)
Th2	T helper 2 (cell)
Th17	T helper 17 (cell)
TLR4	Toll-like receptor 4
Treg	regulatory T (cell)
WBC	white blood cell
ε^2^	epsilon-squared (Kruskal–Wallis effect size)
